# Measurable residual disease (MRD)-testing in haematological and solid cancers

**DOI:** 10.1038/s41375-024-02252-4

**Published:** 2024-04-18

**Authors:** Junren Chen, Robert Peter Gale, Yu Hu, Wen Yan, Tiantian Wang, Wei Zhang

**Affiliations:** 1 State Key Laboratory of Experimental Hematology, National Clinical Research Center for Blood Diseases, Haihe Laboratory of Cell Ecosystem, Institute of Hematology & Blood Diseases Hospital, Chinese Academy of Medical Sciences & Peking Union Medical College, Tianjin, China; 2 Tianjin Institutes of Health Science, Tianjin, China; 3 Centre for Haematology, Department of Immunology and Inflammation, Imperial College of Science, Technology and Medicine, London, UK

**Keywords:** Cancer, Medical research

*“When you can measure what you are speaking about and express it in numbers, you know something about it.”*

Lord Kelvin

There is considerable interest in and enthusiasm for quantitative tests for residual cancer cells in the context of cancer therapy, a concept referred to as measurable residual disease (MRD)-testing. However, an updated critical evaluation of using MRD-tests to predict cancer recurrence and to direct subsequent cancer therapy(ies) is needed. We review concepts underlying MRD-testing and results of studies of MRD-testing in haematological and solid cancers. Most important, we examine if there are any convincing data proving therapy decisions in someone with cancer should be guided by results of MRD-testing or a positive MRD-test result provides sufficient and meaningful lead time to intervene and substantially change clinical outcomes.

## Measurable residual disease (MRD)

In the context of cancer, MRD-testing attempts to quantify residual cancer cells when the cancer is no longer detectable by conventional methods including blood tests, biopsy or radiological studies such as ^18^F-deoxyglucose positron emission tomography (PET), computed tomography (CT) and/or magnetic resonance imaging (MRI). The concept of MRD-testing is not new. Since the 1950s blood concentration of beta-human chorionic gonadotropin (β-hCG) has been used to monitor response to chemotherapy of trophoblastic cancers [1]. Other examples include use of blood concentrations of prostate specific antigen (PSA) in prostate cancer, carcinoembryonic antigen (CEA) in colorectal and lung cancers and cancer antigen 125 (CA-125) in ovary cancer.

The best examples of the utility of MRD-testing are in chronic myeloid leukaemia (CML), acute promyelocytic leukaemia (APL) and acute lymphoblastic leukaemia (ALL) because these diseases have well-defined molecular signatures [2–4]. However, these cancers are atypical. CML and APL are caused by a single canonical mutation shared by all affected persons: the *breakpoint cluster region*–*Abelson tyrosine protein kinase-1* (*BCR::ABL1*) and the *promyelocytic leukaemia protein*–*retinoic acid receptor alpha* (*PML::RARA*) fusion genes, respectively. On the other hand, each case of ALL has a unique immunoglobulin (IG) or T-cell receptor (TCR) rearrangement which can serve as a clonal marker. Other cancers might not be as well-defined in DNA or RNA. Quantitative definition for the depth of molecular response is the most standardised in CML [5, 6]. Persons with CML who attain deep molecular response (DMR) may decide to stop taking tyrosine kinase inhibitors (TKIs) [6–11]. For these people, conversion from negative to positive MRD-test results i.e. loss of major molecular response [MMR]) is often a trigger for re-starting TKI-therapy [6–9, 12].

Most studies of MRD are in haematological cancers but there are increasing numbers in solid cancers. Although MRD-tests in haematological cancers and in solid cancers seem similar, there are important fundamental differences. In haematological cancers MRD-testing is usually done in persons achieving a complete remission/response after receiving or completing systemic therapy. In contrast, in solid cancers MRD-testing is sometimes done immediately after surgical resection of a (presumably) localised cancer.

MRD-tests in blood cancers include multi-parameter flow cytometry (MPFC), real-time quantitative reverse transcription PCR (RT-qPCR) or digital PCR (dPCR) of RNA molecules, real-time quantitative PCR (qPCR) or dPCR of DNA molecules and next-generation sequencing (NGS) [13–18]. MRD-testing in solid cancers mostly focuses on identifying cancer cells or DNA from them in blood via targeted detection of cancer-related mutation(s) [19]. There are important differences among various types of assays. MPFC enumerates (mostly) live cells one-by-one [20, 21]. In contrast, NGS detects cell-free DNA (released by normal or cancer cells that undergo apoptosis or necrosis) or DNA extracted from live cells [22, 23]. RT-qPCR assays often implicitly assume all cancer cells have equal transcription rates.

## Accuracy of MRD-testing to predict relapse/recurrence

There is often a correlation between a positive MRD-test result and cumulative incidence of cancer relapse/recurrence (CIR). However, previously-reported numbers were not stellar [24]. For example, in CML a positive MRD-test predicted a 42–74 percent cumulative incidence of cytogenetic or haematological relapse whereas the positive predictive value (PPV) was reported to be <60 percent in ALL and AML [25–29]. The question is how much the field has advanced and currently at what rates of positive and negative predictive values (PPV; NPV) of cancer recurrence?

To address this, we interrogated data from 1510 publications on MRD during 1 January 2013–7 October 2023 in 17 high impact factor medical journals using the Boolean search terms ‘cell free DNA’, ‘cell-free DNA’, ‘cfDNA’, ‘circulating tumour DNA’, ‘ctDNA’, ‘measurable residual disease’, ‘minimal residual disease’, ‘MRD’ and ‘residual disease’ (Supplementary Fig. 1). ‘Circulating tumour cells’ (CTC) was not a search term but some CTC studies were identified by other search terms. Cell-free DNA (cfDNA) is the most commonly studied analyte in liquid biopsies and more commonly used in solid cancers compared with CTC [30]. 25 of the 1510 publications mentioned CTC. We identified 95 articles including 15 from *LEUKEMIA* which studied > 50 subjects and had data on relationship between MRD-test results and cumulative incidence of histological relapse or clinical or radiological progression [31–125]. 82 were studies in haematological cancers and 13 in solid cancers. CML studies that defined relapse as loss of MMR (i.e. molecular relapse) were not included [126–128]. If definition of relapse is a previously negative MRD-test becoming positive or a previously weakly-positive MRD-test becoming stronger, naturally there is an absolute correlation between MRD-test results and relapse, a self-fulfilling prophesy.

For 79 articles we were able to calculate the odds ratio (OR) of CIR between subjects with positive and negative MRD-tests receiving the same therapy (Table 1; Supplementary Table 1) [31–35, 38–44, 46–49, 53–69, 71–74, 76–79, 82–91, 95–99, 101–110, 113–125]. Median study cohort size was 147 subjects (inter-quartile range [IQR], 86–224) for haematological cancers and 77 (IQR, 59–112) for solid cancers. AML (*N* = 38) and ALL (*N* = 23) were the most commonly studied haematological cancers and colorectal (*N* = 5) and breast (*N* = 4) cancers, the most commonly studied solid cancers. In studies of haematological cancers the most commonly studied MRD-test time points were during/after remission induction (*N* = 33) and pretransplant (*N* = 24). In studies of solid cancers the most common MRD-test time point was immediately post-resection (*N* = 8). In haematological cancers the most commonly studied MRD-test assays were MPFC (*N* = 31) and PCR (*N* = 24) whilst in solid cancers NGS (*N* = 10) was the most common MRD-assay.

**Table 1:** Articles that studied the association of MRD-test results with relapse risk in persons receiving identical therapy.

Sub-group	Articles, *N* (%)
Publication year	
Haematological cancers	66 (84)
Before 31 Dec 2018	33 (42)
After 1 Jan 2019	33 (42)
Solid cancers	13 (16)
After 1 Jan 2019	13 (16)
Disease	
Haematological cancers	66 (84)
ALL	23 (29)
AML	38 (48)
Lymphoma	2 (3)
MM	1 (1)
>1 cancer types	2 (3)
Solid cancers	13 (16)
Patient age	
Haematological cancers	66 (84)
Adults	39 (49)
Children	11 (14)
Adults + Children	15 (19)
NA	1 (1)
Solid cancers	13 (16)
Adults	13 (16)
MRD-test time	
Haematological cancers	66 (84)
During or after induction	33 (42)
During or after consolidation	9 (11)
Before transplant	24 (30)
After transplant	9 (11)
End of treatment	2 (3)
Solid cancers	13 (16)
Before surgery	3 (4)
After surgery	8 (10)
After adjuvant chemotherapy	2 (3)
End of treatment	2 (3)
MRD-test assay	
Haematological cancers	66 (84)
MPFC	31 (39)
PCR	24 (30)
NGS	8 (10)
>1 assay types	6 (8)
NA	1 (1)
Solid cancers	13 (16)
PCR	3 (4)
NGS	10 (13)
CTC	1 (1)
>1 assay types	1 (1)

*ALL* acute lymphoblastic leukaemia, *AML* acute myeloid leukaemia, *CTC* circulating tumour cell enumeration, *MM* multiple myeloma, *MPFC* multi-parameter flow cytometry, *MRD* measurable residual disease, *NA* not applicable, *NGS* next-generation sequencing, *PCR* polymerase chain reaction.

Not all studies reported estimated standard errors of CIR, PPV or NPV. However, the standard error of the logarithm of OR for likelihood of relapse/recurrence in subjects with a positive MRD-test compared with those with a negative MRD test should be approximately proportional to $1/\sqrt{N}$, where *N* is the cohort size [129]. Based on this assumption we used Egger regression to correct for variation in cohort size across the studies and detect plausible publication bias [130]. We detected no publication bias (Supplementary Fig. 2). After correcting for variation in cohort size the estimated average OR for likelihood of relapse/recurrence in subjects with positive MRD compared with those with negative MRD was OR = 3.5 (95% confidence interval [CI], [2.3, 5.4]) in haematological and 9.1 ([3.3, 24.9]) in solid cancers (Table 2). The greater accuracy of MRD-testing of blood samples in predicting relapse/recurrence in solid cancers is possibly because detection in blood samples implies these persons may already have metastases.

**Table 2:** Sub-group analysis of the odds ratio of relapse risk between subjects with positive and negative MRD-tests.

Sub-group	Odds ratio (95-percent CI)^a^
Haematological cancers	
All (*N* = 66)	3.5 (2.3–5.4)
Publication year	
Before 31 Dec 2018 (*N* = 33)	2.3 (1.0–5.4)
After 1 Jan 2019 (*N* = 33)	4.5 (2.4–8.3)
Cancer type	
ALL (*N* = 23)	2.5 (1.3–4.5)
AML (*N* = 38)	4.7 (2.6–8.6)
Patient age	
Adults (*N* = 39)	4.4 (2.5–7.6)
Children (*N* = 11)	1.7 (0.3–9.8)
MRD-test time	
During or after induction (*N* = 33)	1.6 (1.0–2.6)
During or after consolidation (*N* = 9)	12.8 (5.8–28.6)
Before transplant (*N* = 24)	6.3 (3.7–10.6)
After transplant (*N* = 9)	21.0 (6.8–65.3)
MRD-test assay	
MPFC (*N* = 31)	4.4 (2.4–8.1)
PCR (*N* = 24)	2.2 (0.5–9.4)
NGS (*N* = 8)	4.9 (1.1–22.1)
Solid cancers (*N* = 13)	9.1 (3.3–24.9)

*ALL* acute lymphoblastic leukaemia, *AML* acute myeloid leukaemia, *CI* confidence interval, *MPFC* multi-parameter flow cytometry, *MRD* measurable residual disease, *NGS* next-generation sequencing, *PCR* polymerase chain reaction.

^a^Estimated by Egger regression.

In our analysis we compared highly diverse MRD-test targets (specific cancer markers like *BCR::ABL1*, clonal markers such as IG/TCR rearrangements, aberrant cell phenotypes, circulating tumour DNA [ctDNA] etc.), assay types (MPFC, PCR, NGS etc.) and cancers, raising the question whether it is legitimate to consider these together. Consequently, we also did sub-group analyses of haematological cancers focusing on different publication periods, types of leukaemia, MRD-testing time points and MRD-test assays (Table 2). MRD-tests in AML had higher ORs compared with ALL (4.7 [2.6, 8.6] versus 2.5 [1.3, 4.5]). MRD-tests done during/after consolidation chemotherapy had higher ORs compared with MRD-tests during/after remission induction (12.8 [5.8, 28.6] versus 1.6 [1.0, 2.6]). Finally, MRD-tests done posttransplant had higher ORs compared with tests done pretransplant (21.0 [6.8, 65.3] versus 6.3 [3.7, 10.6]). When done at selective time points (i.e. during/after consolidation chemotherapy or after transplant), accuracy of MRD-tests for predicting CIR in haematological cancers exceeds accuracy in solid cancers. However, the later we do MRD-testing the less likely it is a subsequent intervention will change outcomes. For example, a positive MRD-test result posttransplant by itself is unlikely to change subsequent therapy. There is also the issue of the interval between a positive MRD-test and clinical relapse as the lead time might not be meaningful.

Despite the high ORs of positive MRD-test results for predicting CIR, PPVs were highly variable (Table 3). MRD-tests are useful for identifying sub-cohorts with different CIRs (i.e. risk-stratification). However, MRD-testing is less accurate if we want to identify which persons in a cohort will relapse or not. Median PPV was only 55 percent in haematological (IQR, 40–70%) and 75 percent in solid cancers (IQR, 56–77%). Median NPV was 77 percent in haematological (IQR, 69–86%) and 88 percent in solid cancers (IQR, 83–92%). For haematological cancers, PPVs ranged from 41 percent in children or ALL to 73 percent for tests done posttransplant whereas NPVs ranged from 71 percent for tests done during/after consolidation chemotherapy to 83 percent for tests done posttransplant. Even in the best-case scenario (i.e. MRD-tests done posttransplant) median PPVs and NPVs were unsatisfactory. However, our analyses share weaknesses with other similar analyses [131, 132].

**Table 3:** Sub-group analysis of positive and negative predictive values of a positive MRD-test.

Sub-group	Positive predictive value %, median (IQR)	Negative predictive value %, median (IQR)
Haematological cancers		
All (*N* = 66)	55 (40–70)	77 (69–86)
Publication year		
Before 31 Dec 2018 (*N* = 33)	61 (46–78)	75 (69–82)
After 1 Jan 2019 (*N* = 33)	50 (35–62)	79 (72–93)
Cancer type		
ALL (*N* = 23)	41 (27–60)	82 (75–93)
AML (*N* = 38)	65 (50–75)	75 (64–82)
Patient age		
Adults (*N* = 39)	62 (48–72)	74 (66–79)
Children (*N* = 11)	41 (35–60)	82 (75–92)
MRD-test time		
During or after induction (*N* = 33)	55 (36–75)	74 (65–87)
During or after consolidation (*N* = 9)	61 (52–67)	71 (65–77)
Before transplant (*N* = 24)	53 (38–65)	79 (75–82)
After transplant (*N* = 9)	73 (46–86)	83 (76–89)
MRD-test assay		
MPFC (*N* = 31)	58 (41–74)	75 (63–88)
PCR (*N* = 24)	55 (40–71)	77 (72–86)
NGS (*N* = 8)	66 (53–68)	76 (73–83)
Solid cancers (*N* = 13)	75 (56–77)	88 (83–92)

*ALL* acute lymphoblastic leukaemia, *AML* acute myeloid leukaemia, *IQR* inter-quartile range, *MPFC* multi-parameter flow cytometry, *MRD* measurable residual disease, *NGS* next-generation sequencing, *PCR* polymerase chain reaction.

## Sources of errors

Accuracy of MRD-testing in predicting outcomes varies and correlates with cancer type, assay type, how representative a sample used for MRD-testing is of residual cancer cells and other parameters [19]. Considering the extensive data from MRD-testing in haematological cancers it is important to review lessons learned and implications.

MRD-test results are quantitative, but a clinical decision is usually made based on applying one or more pre-defined thresholds to the MRD-test results. Therefore, MRD-test results are often used as binary (negative/positive) or ternary (negative/weak/strong). However, a fixed set of cut-off threshold values fail to reflect different kinetics of wide-ranging leukaemia sub-types. For example, an MRD concentration of 0.01 percent in childhood ALL with high-risk genetics (e.g. *lysine methyltransferase 2A* (*MLL*) fusions) has the same relapse risk as an MRD concentration of 1 percent in children with hyper-diploidy [133]. Consequently, cut-off thresholds for MRD-test results should reflect biological features of the cancer being considered, but this is rarely so in practice. Also, sensitivity and specificity of MRD-tests are evolving over time and variable across assays.

In childhood ALL it is common to use sequential MRD-testing to estimate relapse risk and adjust therapy-intensity accordingly [75, 94]. This dynamic, adaptive approach is not standardised in most other cancers save CML where dose and/or type of TKI therapy is adjusted based on results of sequential MRD-testing for *BCR::ABL1*. Diverse leukaemia types have different proliferation rates, sensitivities to chemotherapy and/or biological features including the likelihood of causing relapse which need to be accounted for. For example, it is reportedly necessary to do MRD-testing every 2 months in AML with the *PML::RARA* fusion gene to reliably detect MRD with 90-percent confidence and >2-month lead time before histological relapse [134]. In contrast, some data suggest monthly MRD-testing may be necessary in AML with the *core-binding factor subunit beta*–*myosin heavy chain 11* (*CBFB::MYH11*) fusion gene to achieve this goal [90].

Another important limitation of MRD-testing is that not all leukaemia cells detected have the biological ability to cause relapse [135, 136]. Presently, there is no reliable way to distinguish cells with and without this ability. Competing causes of death in persons with leukaemia further complicate evaluating accuracy of MRD-testing. For example, someone with a positive MRD-test may die from an unrelated cause before relapse resulting in a seemingly false-positive test result when the outcome measure is leukaemia-free survival or survival rather than CIR [137]. Other endpoints such as time-to-next-therapy (TTNT) are also inappropriate because of subjectivity in deciding whom to treat and when.

One important cause of false-negative MRD-tests is that a sample may not be representative of the number of cancer cells in someone. This limitation is perhaps as or more important compared with sensitivity and specificity of the MRD-assay. Blood and bone marrow are easily sampled but it is often incorrectly assumed the distribution of cancer cells in these samples is representative of the distribution of cancer cells throughout the body. Bone marrow is often the default sample for MRD-testing in haematological cancers although a blood sample is a similarly or even more accurate relapse predictor in some settings [53, 54, 69, 138, 139]. Obviously, a sample containing no cancer cell cannot be informative regardless of assay sensitivity when the assay is based on analysing intact cells or RNA or DNA extracted from intact cells. In chronic lymphocytic leukaemia (CLL), for example, residual leukaemia cells in spleen and lymph nodes are unlikely to be identified in MRD-tests done on blood or bone marrow samples, a limitation that may be overcome by testing for cfDNA [140]. The same limitation applies to plasma cell myeloma where bone marrow involvement is often spatially heterogeneous. This limitation also applies to multi-site involvement in lymphomas. These situations parallel the concept of testing blood samples in persons with solid cancers where there may be undetectable metastases at distant sites. For example, breast cancer brain metastases are less likely to be detected by an MRD-test of a blood sample [113]. Even without spatial heterogeneity there is Poisson noise. The number of leukaemia cells in a 5 ml sample from a 5 L blood volume fluctuates as a result of Poisson noise alone and taking this into account improves interpretation of MRD tests [141, 142]. With the proviso you have a sensitive and specific assay, cell-free detection of ctDNA in solid cancers might be less susceptible to Poisson noise than other MRD-testing assays that rely on presence of intact cancer cells in a sample (e.g. detection of *BCR::ABL1* transcripts in leukaemia cells). When the unit of detection is numbers of molecules rather than cells Poisson noise is less of a concern if molecule concentrations are orders of magnitude larger than cell concentrations.

Another cause of false-negative MRD-tests is the considerable phenotype and genotype heterogeneity of cancer cells [143, 144]. For example, in CML some leukaemia cells carry a newly-mutated, resistant *BCR::ABL1* fusion gene but mutated transcripts are undetectable by NGS [145]. Consequently, testing for mutated *BCR::ABL1* transcripts may not identify all resistant leukaemia cells. Moreover, residual leukaemia cells can be in different immune-phenotype-defined sub-populations with considerable variation among people [146, 147]. In many cancers new sub-clones may emerge spontaneously or in response to therapy. This makes MRD-testing an exercise of chasing a moving target [148, 149].

The technology used for MRD-testing should also be considered when interpreting results. ctDNA is derived from cancer cells which have died from apoptosis or necrosis spontaneously or as a result of therapy [22, 23, 150]. Consequently, quantification of ctDNA might not reflect numbers of residual, live cancer cells. Nor can it distinguish therapy-sensitive and -resistant cells. Guidelines for MPFC-based MRD-testing often recommend declaring a test positive only if ≥5 × 10E+5 cells are analysed and if ≥20 or ≥50 cells are positive [14, 78, 151]. Adopting these guidelines decreases false-positives but increases false-negatives [141, 152]. Using fusion gene transcript concentration as a proxy for numbers of residual cancer cells implicitly assumes all cancer cells have equal transcription rates. However, a recent study in CML reported discordance between sizes of resistant sub-clones of leukaemia cells estimated by quantifying DNA versus RNA transcripts in many persons [145]. Presently, it is unclear if any technology is better than others for MRD-testing. With ‘next-generation flow cytometry’, MPFC is able to achieve a sensitivity of 2 × 10E–6 and is reportedly more accurate compared with NGS-based MRD-testing for predicting posttransplant PFS and survival in plasma cell myeloma [153].

In summary, data from studies in haematological cancers indicate MRD-testing is useful for predicting relapse risk but use of MRD-test results is complex and with substantial limitations resulting in high false-positive and -negative rates. Some of these limitations are potentially surmountable (e.g. adjusting the frequency of MRD-testing) whereas others, such as Poisson noise, are more difficult to overcome.

## Should we use results of MRD-testing to guide subsequent therapy?

Given MRD-tests’ inherent limitations we interrogated data to determine how useful are results of MRD-testing to guiding subsequent therapy(ies) such as intensifying therapy (e.g. a haematopoietic cell transplant) or withholding therapy (e.g. adjuvant chemotherapy in resected colorectal cancer).

Only 18 of the 95 articles in our literature survey had data on the effect of MRD-guided therapy on relapse risk. All were limited to acute leukaemias (Table 4). Five concluded it is possible to withhold therapy in MRD-test-negative subjects without increasing relapse risk whereas 1 concluded otherwise. In contrast, 7 concluded it is possible to reduce relapse risk by intensifying therapy in MRD-test-positive subjects whereas 6 concluded it is not.

**Table 4:** Is it justified to tailor treatment according to MRD-test results?

Cancer	MRD-test assay	MRD-test time point	Does increasing therapy-intensity lower relapse risk in people with positive MRD-tests? (intervention vs. control)	Does decreasing therapy-intensity increase relapse risk in people with negative MRD-tests? (intervention vs. control)	RCT?	*N*	Publication year	Ref.
Relapsed ALL in children	PCR	End of induction	Yes (27% vs. 59% at 8 years)	–	No	133	2013	[36]
ALL	PCR	Within 11 weeks after therapy start	–	No	Yes	521	2013	[37]
*BCR::ABL1*-positive ALL	PCR or FISH	Before transplant	Yes (35% vs. 61% at 3 years)	–	No	101	2014	[38]
ALL	PCR	29 days after therapy start	Yes (8% vs. 14% at 5 years)	–	Yes	533	2014	[45]
AML	MPFC	Before transplant	No	–	No	51	2015	[50]
*BCR::ABL1*-negative ALL	PCR	After 1 cycle of induction	Yes (HR = 0.39)	–	No	522	2015	[51]
AML	MPFC	After consolidation	Yes (46% vs. 70% at 6 year)	–	No	105	2015	[52]
ALL in children	PCR	Within 12 weeks after therapy start	–	No	Yes	1164	2018	[70]
ALL in children	MPFC	15 days after therapy start	Yes (1.8% vs. 5.7% at 5 years for CNS relapse)	–	No	359	2019	[75]
ALL or AML	PCR, MPFC or NGS	Before transplant	No	–	No	169	2020	[77]
AML	MPFC	Before transplant	No	–	No	79	2020	[80]
AML	NGS	Before transplant	Yes (14% vs. 58% at 1 year)	–	No	125	2020	[81]
B-cell ALL in children	MPFC	29 days after therapy start	–	No	Yes	1857	2021	[92]
High-risk AML or MDS	MPFC	Before transplant	No	–	No	244	2021	[93]
ALL in children	MPFC	Within 46 days after therapy start	–	No	Yes	2923	2021	[94]
ALL	PCR	End of induction	–	Yes (8% vs. 4% at 10 years)	Yes	521	2022	[100]
ALL	PCR	End of induction	No	–	Yes	533	2022	[100]
ALL in children	PCR	End of induction	–	No	No	369	2023	[111]
ALL in children	MPFC	15 days after therapy start	No	–	Yes	892	2023	[112]

*ABL1* Abelson tyrosine protein kinase 1, *ALL* acute lymphoblastic leukaemia, *AML* acute myeloid leukaemia, *BCR* breakpoint cluster region, *CNS* central nervous system, *FISH* fluorescence in situ hybridisation, *HR* hazard ratio, *MDS* myelodysplastic syndromes, *MPFC* multi-parameter flow cytometry, *MRD* measurable residual disease, *NGS* next-generation sequencing, *PCR* polymerase chain reaction, *RCT* randomised controlled trial.

**Fig. 1:**
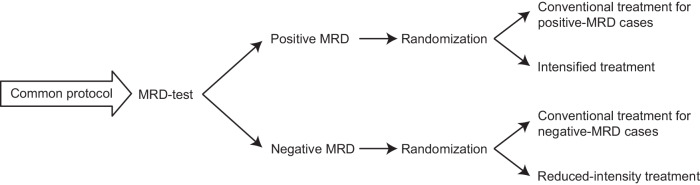
Optimal design of a randomised controlled trial that tests the efficacy of MRD-guided therapy.

Ideally, studies testing the efficacy of MRD-testing should be RCTs. An optimal RCT for this purpose would be a 2 × 2 design and treat the entire cohort of subjects on the same protocol until an MRD-test is done and then randomise the subjects into two control arms (i.e. conventional therapy[ies] for positive- and negative-MRD subjects) and two experimental arms (i.e. experimental therapy[ies] for positive- and negative-MRD subjects) (Fig. 1). This was rarely done. Only 7 articles in our literature survey were bona fide RCTs, all limited to ALL in children and/or young adults. In children with ALL, early response to remission induction therapy including results of MRD-testing is often used to classify children into different ‘risk’ strata and then channel them into different therapy-intensity paths. Once this is done the results of post-remission induction MRD-testing are usually no longer used to guide therapy. This paradigm of ‘early response-guided therapy’ or ‘MRD-guided therapy’ was not originally developed based on data from RCTs but is associated with recent survival improvements [154–159]. In 7 studies in our survey RCTs tested the possibility of decreasing therapy-intensity *further* in low-risk children or increasing therapy-intensity *further* in high-risk children [37, 45, 70, 92, 94, 100, 112]. Five articles described RCTs of whether decreasing therapy-intensity in MRD-test-negative subjects increased CIR; 4 concluded no. Three articles described RCTs of whether increasing therapy-intensity in MRD-test-positive children decreased CIR; only 1 concluded yes. Ongoing RCTs on MRD-guided interventions in solid cancers are reviewed elsewhere; results are pending [160].

In summary, it is unclear if we can reduce relapse/recurrence risk through positive-MRD-guided intervention or withhold therapy based on negative MRD-test results in most cancers. There is reasonably strong support for decreasing therapy-intensity in MRD-test-negative children and/or young adults with ALL whilst data are lacking for other cancers such as AML and CLL. In contrast, intensifying treatment in a person with a positive MRD-test might temporarily drive MRD-test results to negative but often this is not correlated with a lower CIR or better survival. For example, some haematologists argue people with AML and a positive MRD-test should receive a haematopoietic cell transplant whereas others argue the contrary because transplant outcomes in these people are poor [104, 161]. Efficacy can only be proven in an RCT but such trials are infrequently done.

**Fig. 2:**
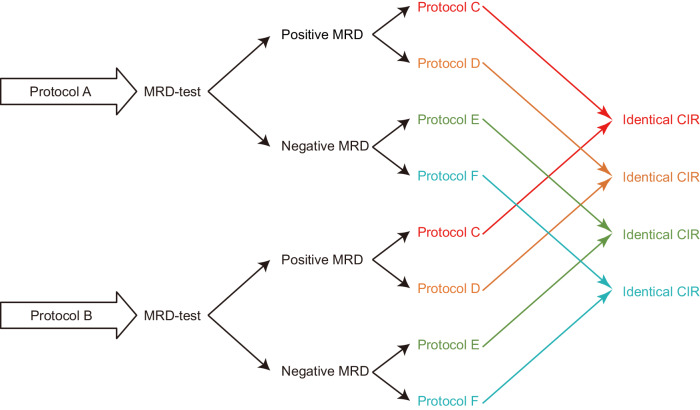
Conditions that need to be met for MRD state to be a *perfect* surrogate for relapse risk when running clinical trials to compare efficacy of protocols. To be able to use MRD state as a surrogate for cumulative incidence of relapse (CIR) when comparing protocols in clinical trials, it is crucial that MRD-test results have the same implications for CIR and follow-up interventions (or lack thereof) regardless of which protocol (A versus B) is used prior to the MRD-test. More explanations are available in Supplementary Methods.

Conjoint analysis of RCTs of MRD-guided therapies will also help us decide if MRD state can be used as a surrogate for CIR in future clinical trials. MRD-test results positively correlate with CIR at the sub-cohort level (albeit imperfectly) as we discuss above. However, this correlation alone is insufficient. If MRD state is indeed a *perfect* surrogate for CIR a positive MRD-test should imply the same CIR regardless of the treatment protocol used prior to the MRD-test (Fig. 2; Supplementary Methods). The true endpoint rate at any follow-up time (i.e. CIR) should be independent of prior therapy given the values of the surrogate variable (i.e. MRD-test results) [162]. Otherwise, it would be premature to declare a negative MRD-test a success and a positive MRD-test a failure, because positive MRD has different meanings in persons with the same disease treated on different protocols and in some (but not all) protocols CIR may still be modifiable by adjusting subsequent therapy. Presently, MRD state has not satisfied the stringent operational criteria for a surrogate endpoint.

## Conclusion

Categorising MRD-test results into binary or ternary is an efficient way to facilitate rapid decision-making. However, this di- or trichotomization might delude us into believing there is a solid foundation underlying our decisions for MRD-guided interventions in most cancers. This is not so.

Our analyses suggest there has been too little focus on *therapeutic* implications of MRD-test results. Only RCTs can definitively prove whether an intensified intervention in people who are MRD-test-positive improves outcomes compared with conventional management. Similarly, only RCTs can prove whether withholding an intervention in a person who is MRD-test-negative is without risk.

Finally, it is important to recognise that a positive MRD-test after a therapy intervention might identify people with biologically more aggressive cancers compared with those with a negative MRD-test and that cancer cells detected by the MRD-test might not be the cause of the increased CIR but be merely assocated with it.

### Supplementary information

SUPPLEMENTARY
