# Supplementary material for: Measurable residual disease (MRD)-testing in haematological and solid cancers
Source: Leukemia. 2024 Apr 18;38(6):1202–12. doi: 10.1038/s41375-024-02252-4 (PMC11147778; doi:10.1038/s41375-024-02252-4)
Supplement: Supplementary file 1 — SUPPLEMENTARY [file 41375_2024_2252_MOESM1_ESM.docx]

**Supplement Information**

Measurable residual disease (MRD)-testing in haematological and solid cancers

Junren Chen^1,2^, Robert Peter Gale^3^, Yu Hu^1,2^, Wen Yan^1,2^, Tiantian Wang^1,2^, Wei Zhang^1,2^

^1^ State Key Laboratory of Experimental Hematology, National Clinical Research Center for Blood Diseases, Haihe Laboratory of Cell Ecosystem, Institute of Hematology & Blood Diseases Hospital, Chinese Academy of Medical Sciences & Peking Union Medical College, Tianjin, China

^2^ Tianjin Institutes of Health Science, Tianjin, China

^3^ Centre for Haematology, Department of Immunology and Inflammation, Imperial College of Science, Technology and Medicine, London, UK

**Correspondence**

Junren Chen MD PhD

Institute of Hematology & Blood Diseases Hospital

Chinese Academy of Medical Science

Tianjin 300020, China

E [chenjunren@ihcams.ac.cn](mailto:chenjunren@ihcams.ac.cn)

**Supplement Table 1. How prognostic are MRD-test results of relapse/recurrence risk?**

| **Cancer** | **MRD-test assay** | **MRD-test time point** | **Treated on the identical protocol** | | ***N*** | **Publication year** | **Ref.** |
| --- | --- | --- | --- | --- | --- | --- | --- |
|  |  |  | **CIR in positive MRD-test cases** | **CIR in negative MRD-test cases** |  |  |  |
| AML | MPFC | CR after 1 cycle of induction | ≈48% at 3 years | ≈28% at 3 years | 164 | 2013 | (1) |
| AML | MPFC | CR after 2 cycles of induction | ≈75% at 3 years | ≈38% at 3 years | 183 | 2013 | (1) |
| AML | MPFC | CR after consolidation | ≈68% at 3 years | ≈26% at 3 years | 121 | 2013 | (1) |
| AML | MPFC | CR1 before transplant | 58% at 3 years | 21% at 3 years | 183 | 2013 | (2) |
| AML | MPFC | CR2 before transplant | 68% at 3 years | 19% at 3 years | 70 | 2013 | (2) |
| AML in older persons | MPFC | CR after 1 cycle of treatment | 83% at 3 years | 71% at 3 years | 286 | 2013 | (3) |
| AML in older persons | MPFC | CR after 2 cycles of treatment | 91% at 3 years | 79% at 3 years | 279 | 2013 | (3) |
| *NPM1*-mutated AML | PCR | After chemotherapy and/or autologous hematopoietic cell transplant | ≈100% at 3 years | ≈38% at 3 years | 71 | 2013 | (4) |
| *NPM1*-mutated AML | PCR | After allogeneic hematopoietic cell transplant | ≈22% at 3 years | ≈11% at 3 years | 88 | 2013 | (4) |
| *BCR::ABL1*-positive ALL | MPFC | 3 months after CR | ≈55% at 4 years | ≈25% at 4 years | 51 | 2013 | (5) |
| *BCR::ABL1*-positive ALL | PCR or FISH | Before transplant with MAC | 35% at 3 years | 21% at 3 years | 120 | 2014 | (6) |
| *BCR::ABL1*-positive ALL | PCR or FISH | Before transplant with RIC | 61% at 3 years | 31% at 3 years | 65 | 2014 | (6) |
| Mantle cell lymphoma | PCR | After transplant | 67% | 18% | 107 | 2014 | (7) |
| *NPM::ALK*-positive anaplastic large-cell lymphoma in children | PCR | After 1 cycle of induction | 81% at 5 years | 31% at 5 years | 52 | 2014 | (8) |
| ALL | PCR | 6 weeks after therapy start | 60% at 5 years | 23% at 5 years | 354 | 2014 | (9) |
| *NPM1*-mutated AML | PCR | End of induction | 78% at 2 years | 26% at 2 years | 86 | 2014 | (10) |
| *NPM1*-mutated AML | PCR | End of consolidation | ≈70% at 4 years | ≈55% at 4 years | 57 | 2014 | (10) |
| *RUNX1::RUNX1T1*-positive AML | PCR | Before transplant | 24% at 2 years | 6% at 2 years | 92 | 2014 | (11) |
| *RUNX1::RUNX1T1*-positive AML | PCR | 1 month after transplant | 33% at 2 years | 17% at 2 years | 91 | 2014 | (11) |
| *RUNX1::RUNX1T1*-positive AML | PCR | 2 months after transplant | 100% at 2 years | 9% at 2 years | 90 | 2014 | (11) |
| *RUNX1::RUNX1T1*-positive AML | PCR | 3 months after transplant | 46% at 2 years | 11% at 2 years | 86 | 2014 | (11) |
| High-risk ALL in children | MPFC | Before transplant | ≈60% at 2 years | ≈26% at 2 years | 92 | 2014 | (12) |
| Childhood high-risk ALL after first relapse | PCR | End of induction | 61% at 6 years | 34% at 6 years | 119 | 2015 | (13) |
| Childhood high-risk ALL after first relapse | PCR | Before transplant | 49% at 6 years | 32% at 6 years | 71 | 2015 | (13) |
| ALL | NGS | Within 1 year post-transplant | 73% | 13% | 53 | 2015 | (14) |
| Relapsed ALL in children | PCR | Before transplant | 38% at 3 years | 25% at 3 years | 81 | 2015 | (15) |
| Relapsed ALL in children | PCR | 30 days after transplant | 50% at 3 years | 27% at 3 years | 81 | 2015 | (15) |
| Relapsed ALL in children | PCR | 60 days after transplant | 75% at 3 years | 23% at 3 years | 67 | 2015 | (15) |
| Relapsed ALL in children | PCR | 90 days after transplant | 100% at 3 years | 25% at 3 years | 83 | 2015 | (15) |
| Relapsed ALL in children | PCR | 180 days after transplant | 100% at 3 years | 18% at 3 years | 54 | 2015 | (15) |
| AML | MPFC | End of induction | ≈75% at 5 years | ≈39% at 5 years | 245 | 2015 | (16) |
| Standard-risk AML | PCR | After 2 cycles of induction | 82% at 3 years | 30% at 3 years | 194 | 2016 | (17) |
| *RUNX1::RUNX1T1*-positive AML | PCR | End of consolidation | 51% at 4 years | 24% at 4 years | 67 | 2016 | (18) |
| AML | MPFC | Before transplant | 67% at 3 years | 22% at 3 years | 311 | 2016 | (19) |
| AML | MPFC | End of induction | 89% at 1 years | 29% at 1 years | 55 | 2016 | (20) |
| AML | MPFC | Before transplant | ≈74% at 8 years | ≈29% at 8 years | 279 | 2016 | (21) |
| AML | MPFC | ≈28 days after transplant | ≈81% at 8 years | ≈39% at 8 years | 279 | 2016 | (21) |
| AML | MPFC | Before transplant | 33% at 1 year | 14% at 1 year | 152 | 2017 | (22) |
| AML | MPFC | Before transplant | 30% | 8% | 224 | 2017 | (23) |
| AML | PCR | Before transplant | 42% | 21% | 224 | 2017 | (23) |
| *NPM1*-mutated AML | PCR | End of induction | 66% at 3 years | 21% at 3 years | 131 | 2017 | (24) |
| *FLT3*-mutated AML at CR1 or CR2 | PCR | Before transplant | 39% at 2 years | 23% at 2 years | 119 | 2017 | (25) |
| AML in children | MPFC | Before transplant | 38% | 17% | 161 | 2017 | (26) |
| AML in children | MPFC | Within 180 days after transplant | 78% | 14% | 161 | 2017 | (26) |
| AML in older persons | MPFC | CR | 84% at 2 years | 43% at 2 years | 55 | 2018 | (27) |
| AML in older persons | MPFC | 3 months after CR | 86% at 2 years | 48% at 2 years | 61 | 2018 | (27) |
| *BCR::ABL1*-positive ALL in children | PCR (IG/TR) | 33 days after therapy start | 35% at 5 years | 0% at 5 years | 79 | 2018 | (28) |
| *BCR::ABL1*-positive ALL in children | PCR (*BCR::ABL1*) | 33 days after therapy start | 27% at 5 years | 13% at 5 years | 57 | 2018 | (28) |
| AML | NGS | CR | 55% at 4 years | 27% at 4 years | 147 | 2018 | (29) |
| AML | NGS | CR | 46% at 2 years | 24% at 2 years | 122 | 2018 | (30) |
| AML | MPFC | CR | 57% at 2 years | 28% at 2 years | 125 | 2018 | (30) |
| T-cell ALL in children | PCR | CR | 38% at 5 years | 14% at 5 years | 191 | 2018 | (31) |
| Standard-risk AML without *NPM1* mutation | MPFC | CR after 1 cycle of induction | 74% at 4.5 years | 50% at 4.5 years | 203 | 2018 | (32) |
| Standard-risk AML without *NPM1* mutation | MPFC | CR after 2 cycles of induction | 89% at 2.5 years | ≈42% at 2.5 years | 160 | 2018 | (32) |
| Standard-risk AML with *NPM1* mutation | MPFC | CR after 1 cycle of induction | 54% at 5 years | 36% at 5 years | 168 | 2018 | (32) |
| CBF AML | MPFC | CR after 1 cycle of induction | 43% at 5 years | 25% at 5 years | 94 | 2018 | (32) |
| AML | NGS | Before transplant | 66% at 5 years | 17% at 5 years | 96 | 2018 | (33) |
| ALL | MPFC | Before transplant | 31% at 3 years | 16% at 3 years | 543 | 2019 | (34) |
| AML | MPFC | After 2 cycles of induction | ≈60% at 5 years | ≈43% at 5 years | 305 | 2019 | (35) |
| *RUNX1::RUNX1T1*-positive AML | PCR | End of treatment | 55% at 4 years | 23% at 4 years | 86 | 2019 | (36) |
| T-cell ALL | PCR | End of induction | ≈25% at 4 years | ≈5% at 4 years | 145 | 2019 | (37) |
| T-cell ALL | MPFC | End of induction | ≈20% at 4 years | ≈7% at 4 years | 158 | 2019 | (37) |
| AML | MPFC | Before transplant | 66% at 3 years | 22% at 3 years | 606 | 2020 | (38) |
| ALL or AML | PCR, MPFC or NGS | Before transplant | 33% at 2 years | 18% at 2 years | 506 | 2020 | (39) |
| MM | MPFC | End of consolidation | 46% at 3 years | 10% at 3 years | 357 | 2020 | (40) |
| High-risk haematological cancers in children | Not reported | Before transplant | 57% at 2 years | 25% at 2 years | 171 | 2020 | (41) |
| *E2A::PBX1*-positive B-ALL | PCR or MPFC | End of induction | 59% at 5 years | 19% at 5 years | 135 | 2021 | (42) |
| AML | MPFC | CR after 1 cycle of induction | 50% at 3 years | 41% at 3 years | 1076 | 2021 | (43) |
| AML | PCR | Before transplant | 55% at 4 years | 14% at 4 years | 80 | 2021 | (44) |
| Very-low-risk ALL in children | MPFC | 19 days after therapy start | 17% at 5 years | 5% at 5 years | 200 | 2021 | (45) |
| *KMT2A::AFF1*-positive BCP-ALL | Molecular biology approaches or MPFC | Before transplant | 45% at 5 years | 21% at 5 years | 90 | 2021 | (46) |
| Normal-karyotype BCP-ALL | Molecular biology approaches or MPFC | Before transplant | 37% at 5 years | 26% at 5 years | 189 | 2021 | (46) |
| AML | PCR | Before transplant | ≈50% at 3 years | ≈22% at 3 years | 176 | 2021 | (47) |
| B-ALL with *ABL*-class fusions in children | PCR or MPFC | End of induction | 41% at 5 years | 18% at 5 years | 92 | 2021 | (48) |
| AML | NGS | End of induction | 48% at 3 years | 26% at 3 years | 196 | 2021 | (49) |
| AML | NGS | End of consolidation | 53% at 3 years | 36% at 3 years | 127 | 2021 | (49) |
| AML | MPFC | End of induction | 50% at 3 years | 34% at 3 years | 200 | 2021 | (49) |
| AML | MPFC | End of consolidation | 62% at 3 years | 36% at 3 years | 98 | 2021 | (49) |
| CBF AML | PCR | End of consolidation | 61% at 5 years | 34% at 5 years | 101 | 2021 | (50) |
| Low- to medium-risk BCP-ALL | MPFC | 15 days after therapy start | 10% at 5 years | 4% at 5 years | 1164 | 2021 | (51) |
| ALL in children | MPFC | 15 days after therapy start | 11% at 7 years | 3% at 7 years | 287 | 2022 | (52) |
| ALL in children | MPFC | End of induction | 35% at 7 years | 5% at 7 years | 273 | 2022 | (52) |
| ALL | PCR | After 2 cycles of induction | 46% | 30% | 154 | 2022 | (53) |
| B- or T-cell precursor ALL in children | PCR | ≈2 months after therapy start | 40% at 5 years | 7% at 5 years | 207 | 2022 | (54) |
| AML | MPFC | Before transplant | 65% at 3 years | 23% at 3 years | 810 | 2022 | (55) |
| AML | MPFC | After transplant | 90% at 3 years | 27% at 3 years | 810 | 2022 | (55) |
| AML | MPFC (leukaemic cells) | After transplant | 45% at 2 years | 7% at 2 years | 360 | 2022 | (56) |
| AML | MPFC (leukaemia stem cells) | After transplant | 44% at 2 years | 4% at 2 years | 360 | 2022 | (56) |
| B-cell ALL with *KMT2A* rearrangements in children | PCR or MPFC | End of induction | 23% at 5 years | 16% at 5 years | 273 | 2023 | (57) |
| Early thymic precursor (ETP) T-cell ALL | MPFC | End of induction | ≈7% at 5 years | ≈6% at 5 years | 139 | 2023 | (58) |
| Near-ETP T-cell ALL | MPFC | End of induction | 11% at 5 years | 6% at 5 years | 198 | 2023 | (58) |
| Not-ETP T-cell ALL | MPFC | End of induction | 21% at 5 years | 5% at 5 years | 874 | 2023 | (58) |
| AML | MPFC | Before transplant | 67% at 3 years | 23% at 3 years | 506 | 2023 | (59) |
| AML | NGS | Before transplant | 68% at 3 years | 21% at 3 years | 451 | 2023 | (60) |
| *NPM1*-mutated AML | PCR | After 4 cycles of induction | 72% at 2 years | 10% at 2 years | 76 | 2023 | (61) |
| AML | NGS | CR1 | 68% at 5 years | 13% at 5 years | 62 | 2023 | (62) |
| ALL | MPFC | Before transplant | ≈55% at 3 years | ≈32% at 3 years | 150 | 2023 | (63) |
| B-cell precursor ALL with *KMT2A* rearrangements | PCR | End of induction | ≈62% at 4 years | ≈24% at 4 years | 78 | 2023 | (64) |
| B-cell precursor ALL with *KMT2A* rearrangements | PCR | End of consolidation | ≈70% at 4 years | ≈32% at 4 years | 74 | 2023 | (64) |
| AML with *KMT2A* rearrangements in children | MPFC | After 2 cycles of induction | 65% at 5 years | 46% at 5 years | 418 | 2023 | (65) |
| *FLT3*-ITD AML | NGS | CR | 75% at 4 years | 33% at 4 years | 161 | 2023 | (66) |
| Early-stage breast cancer | PCR | Any time after surgery | 56% (9/16) | 4% (3/85) | 101 | 2019 | (67) |
| Urothelial bladder carcinoma | NGS | Before surgery | 75% (6/8) | 11% (6/55) | 64 | 2019 | (68) |
| Urothelial bladder carcinoma | NGS | After surgery | 76% (13/17) | 0% (0/47) | 64 | 2019 | (68) |
| Non-metastatic colo-rectal cancer | NGS | After surgery | 77% (10/13) | 0% (0/45) | 58 | 2019 | (69) |
| Stage III melanoma | PCR | After surgery | 100% (13/13) | 41% (16/39) | 52 | 2019 | (70) |
| Stage II – III colo-rectal cancer | NGS | Before adjuvant chemotherapy | 70% (7/10) | 12% (5/42) | 52 | 2019 | (71) |
| Stage II – III colo-rectal cancer | NGS | After adjuvant chemotherapy | 100% (7/7) | 14% (7/51) | 58 | 2019 | (71) |
| Stage III colon cancer | NGS | After surgery | 50% (10/20) | 18% (14/76) | 96 | 2019 | (72) |
| Triple-negative breast cancer | NGS | After neoadjuvant chemotherapy | 35% (23/66) | 13% (6/46) | 112 | 2020 | (73) |
| Triple-negative breast cancer | CTC enumeration | After neoadjuvant chemotherapy | 39% (18/46) | 17% (11/66) | 112 | 2020 | (73) |
| Triple-negative breast cancer | NGS or CTC enumeration | After neoadjuvant chemotherapy | 48% (15/31) | 17% (14/81) | 112 | 2020 | (73) |
| Neoadjuvant-treated breast cancer | NGS | Before surgery | 83% (5/6) | 10% (6/58) | 64 | 2021 | (74) |
| Esophageal adenocarcinomas | NGS | After surgery | 75% (12/16) | 39% (24/61) | 77 | 2021 | (75) |
| High-risk stage II – III hormone receptor-positive breast cancer | NGS | > 5 years after diagnosis | 75% (6/8) | 1% (1/75) | 83 | 2022 | (76) |
| Early-stage non-small cell lung cancer | NGS | Within 4 months after end of treatment | 100% (10/10) | 24% (12/49) | 59 | 2022 | (77) |
| Colo-rectal cancer | NGS | 4 weeks after surgery | 61% (115/187) | 10% (81/852) | 1039 | 2023 | (78) |
| Stage I – III colo-rectal cancer | PCR | After adjuvant chemotherapy | 67% (12/18) | 8% (10/131) | 149 | 2023 | (79) |

Abbreviations: *ABL1*, Abelson tyrosine protein kinase 1; *AFF1*, ALF transcription elongation factor 1; *ALK*, anaplastic lymphoma kinase; ALL, acute lymphoblastic leukaemia; AML, acute myeloid leukaemia; BCP, B-cell precursor; *BCR*, breakpoint cluster region; CBF, core binding factor; CIR, cumulative incidence of relapse/recurrence; CR, complete remission; CR1, first complete remission; CR2, second complete remission; CTC, circulating tumour cell; *E2A*, transcription factor 3; FISH, fluorescence *in situ* hybridization; *FLT3*, fms-like tyrosine kinase 3; IG/TR, rearranged immunoglobulin/T-cell receptor genes; ITD, internal tandem duplication; *KMT2A*, lysine methyltransferase 2A; MAC, myelo-ablative conditioning; MM, multiple myeloma; MPFC, multi-parameter flow cytometry; MRD, measurable residual disease; NGS, next-generation sequencing; *NPM*, nucleophosmin; *PBX1*, pre-B-cell leukaemia homeobox transcription factor 1; PCR, polymerase chain reaction; RIC, reduced-intensity conditioning; *RUNX1*, runt-related transcription factor 1; *RUNX1T1*, RUNX1 partner transcriptional co-repressor 1.

**Supplement Figure 1. Literature search flow chart.**


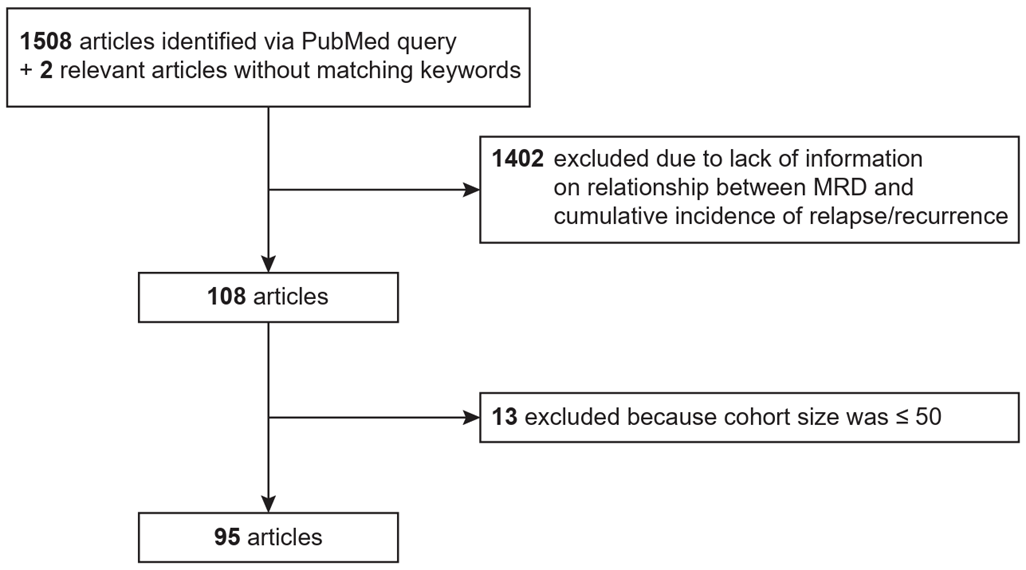


PubMed query: (("Blood"[Journal]) OR ("Haematologica"[Journal]) OR ("Leukemia"[Journal]) OR ("The Lancet. Haematology"[Journal]) OR ("American journal of hematology"[Journal]) OR ("The Lancet. Oncology"[Journal]) OR ("Annals of oncology : official journal of the European Society for Medical Oncology"[Journal]) OR ("Journal of clinical oncology : official journal of the American Society of Clinical Oncology"[Journal]) OR ("JAMA oncology"[Journal]) OR ("Cancer discovery"[Journal]) OR ("Lancet (London, England)"[Journal]) OR ("The New England journal of medicine"[Journal]) OR ("JAMA"[Journal]) OR ("BMJ (Clinical research ed.)"[Journal]) OR ("PLoS medicine"[Journal]) OR ("Nature medicine"[Journal]) OR ("The Journal of clinical investigation"[Journal])) AND (("measurable residual disease") OR ("minimal residual disease") OR ("residual disease") OR ("MRD") OR ("circulating tumor DNA") OR ("ctDNA") OR ("cell-free DNA") OR ("cell free DNA") OR ("cfDNA"))

**Supplement Figure 2. Egger regression to detect publication bias.**

**
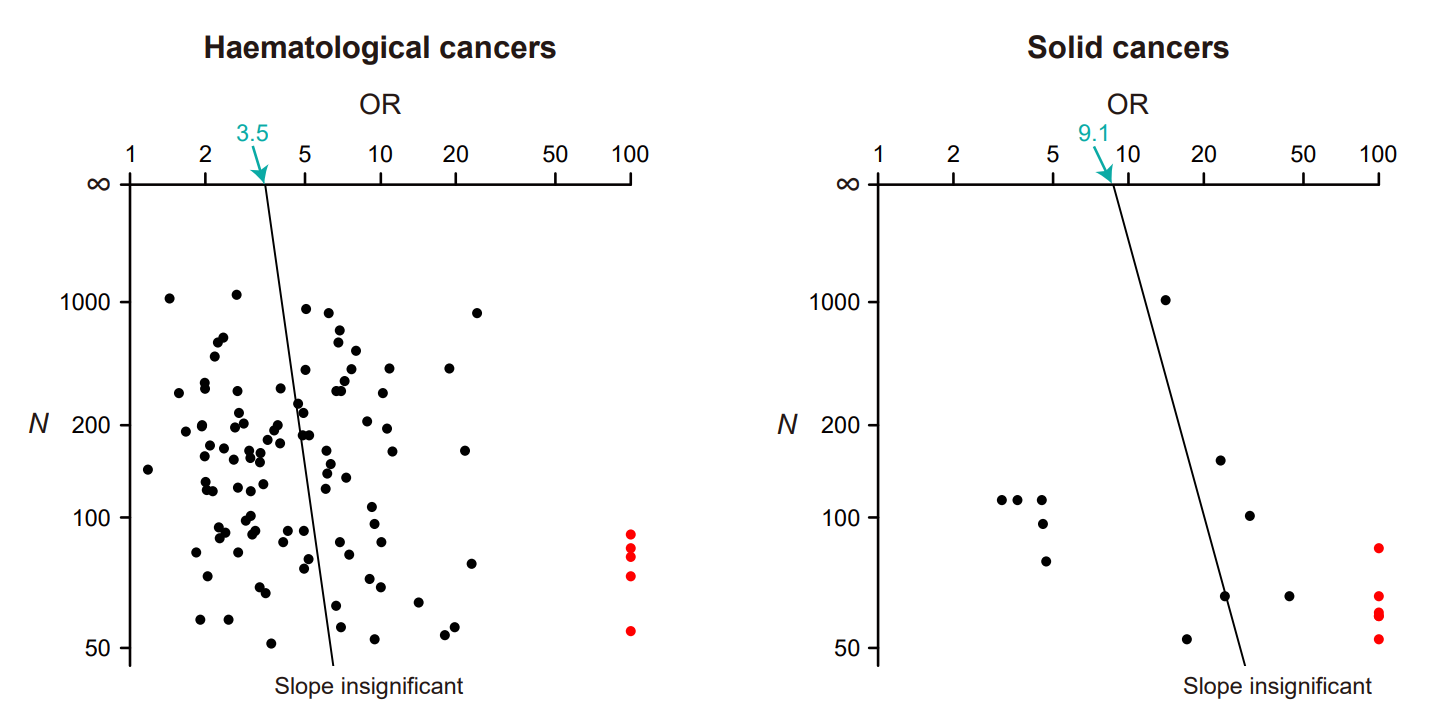
**

Odds is calculated as CIR / (1 – CIR), and odds ratio (OR) as the ratio between odds in the MRD-test-positive cohort and odds in the MRD-test-negative cohort. (When the numerator is infinite or the denominator is zero, OR is set to be 100. Such studies are represented as red dots.) Standard error of the logarithm of OR is proportional to $\frac{1}{\sqrt{N}}$.(80) Cohort size *N* is plotted in $\frac{1}{\sqrt{N}}$ scale and OR in logarithmic scale. Egger regression is conducted as described, using *N* as weights.(81) A regression line that is significantly deviating from vertical suggests publication bias.

**Supplement Methods**

*Conditions that need to be met for MRD state to be a perfect surrogate for relapse risk when running clinical trials to compare efficacy of protocols*

Our derivation is an extension of Prentice (1989).(82)

Let *s* denote the time of *molecular* relapse. Let *t* denote the time of MRD-testing. $s\leq t$ implies a positive MRD-test at time *t* whilst $s>t$ implies a negative MRD-test at time *t*. *k* denotes the treatment protocol used prior to MRD-testing. Let $P_{k}\left( s\leq t \right)$ denote the probability of a positive MRD-test at time *t* conditional on *k* and $P_{k}\left( s>t \right)$, the probability of a negative MRD-test at time *t* conditional on *k*.

Let *q* denote the protocol used after a positive MRD-test and *r*, the protocol used after a negative MRD-test. Assume $T>t$. Let $\lambda_{k}\left( T|q,r \right)$ denote the cumulative incidence of *clinical* relapse (CIR) at time *T* conditional on *k*, *q* and *r*. $\lambda_{k}\left( T|q;s\leq t \right)$ denotes the CIR at time *T* conditional on *k*, a positive MRD-test and *q*. $\lambda_{k}\left( T|r;s>t \right)$ denotes the CIR at time *T* conditional on *k*, a negative MRD-test and *r*.

Therefore,

$$\lambda_{k}\left( T|q,r \right)$$

$$=\lambda_{k}\left( T|q;s\leq t \right)P_{k}\left( s\leq t \right)+\lambda_{k}\left( T|r;s>t \right)P_{k}\left( s>t \right)$$

$=\lambda_{k}\left( T|r;s>t \right)+\left\{ \lambda_{k}\left( T|q;s\leq t \right)-\lambda_{k}\left( T|r;s>t \right) \right\}P_{k}\left( s\leq t \right)$.

Assume we are to conduct clinical trials to compare *m* options of *k*: $k_{1}$, $k_{2}$, …, $k_{m}$. If MRD state is a perfect surrogate for CIR, rankings of $\lambda_{k_{1}}\left( T|q,r \right)$, $\lambda_{k_{2}}\left( T|q,r \right)$, …, $\lambda_{k_{m}}\left( T|q,r \right)$ (*i.e.* rankings of CIR at time *T*) should be identical to rankings of $P_{k_{1}}\left( s\leq t \right)$, $P_{k_{2}}\left( s\leq t \right)$, …, $P_{k_{m}}\left( s\leq t \right)$ (*i.e.* rankings of probabilities of positive MRD at time *t*). Moreover, for ‘negative MRD’ to be a treatment goal in itself, a negative MRD-testing result should imply the same favorable prognosis in every person with the same disease. All of the following three conditions must be met:

1. $\lambda_{k}\left( T|q;s\leq t \right)>\lambda_{k}\left( T|r;s>t \right)$, $\forall k$. That is, regardless of which protocol *k* is used prior to MRD-testing and regardless of which intervention (or lack thereof) after the MRD-test, a positive MRD-test implies a worse CIR than a negative MRD-test. In other words, MRD state should positively correlate with CIR.
2. $\lambda_{k}\left( T|r;s>t \right)$ is independent of *k*. That is, regardless of which protocol *k* is used prior to MRD-testing, a negative MRD-test implies the same CIR provided *r* is fixed.
3. $\lambda_{k}\left( T|q;s\leq t \right)$ is independent of *k*. That is, regardless of which protocol *k* is used prior to MRD-testing, a positive MRD-test implies the same CIR provided *q* is fixed.

Condition 1 is consistent with what are commonly reported in MRD studies. A graphical representation of conditions 2 and 3 is displayed in **Figure 2**.

References

1. Terwijn M, van Putten WL, Kelder A, van der Velden VH, Brooimans RA, Pabst T, et al. High prognostic impact of flow cytometric minimal residual disease detection in acute myeloid leukemia: data from the HOVON/SAKK AML 42A study. J Clin Oncol. 2013;31(31):3889-97.

2. Walter RB, Buckley SA, Pagel JM, Wood BL, Storer BE, Sandmaier BM, et al. Significance of minimal residual disease before myeloablative allogeneic hematopoietic cell transplantation for AML in first and second complete remission. Blood. 2013;122(10):1813-21.

3. Freeman SD, Virgo P, Couzens S, Grimwade D, Russell N, Hills RK, et al. Prognostic relevance of treatment response measured by flow cytometric residual disease detection in older patients with acute myeloid leukemia. J Clin Oncol. 2013;31(32):4123-31.

4. Shayegi N, Kramer M, Bornhauser M, Schaich M, Schetelig J, Platzbecker U, et al. The level of residual disease based on mutant NPM1 is an independent prognostic factor for relapse and survival in AML. Blood. 2013;122(1):83-92.

5. Ravandi F, Jorgensen JL, Thomas DA, O'Brien S, Garris R, Faderl S, et al. Detection of MRD may predict the outcome of patients with Philadelphia chromosome-positive ALL treated with tyrosine kinase inhibitors plus chemotherapy. Blood. 2013;122(7):1214-21.

6. Bachanova V, Marks DI, Zhang MJ, Wang H, de Lima M, Aljurf MD, et al. Ph+ ALL patients in first complete remission have similar survival after reduced intensity and myeloablative allogeneic transplantation: impact of tyrosine kinase inhibitor and minimal residual disease. Leukemia. 2014;28(3):658-65.

7. Kolstad A, Laurell A, Jerkeman M, Gronbaek K, Elonen E, Raty R, et al. Nordic MCL3 study: 90Y-ibritumomab-tiuxetan added to BEAM/C in non-CR patients before transplant in mantle cell lymphoma. Blood. 2014;123(19):2953-9.

8. Damm-Welk C, Mussolin L, Zimmermann M, Pillon M, Klapper W, Oschlies I, et al. Early assessment of minimal residual disease identifies patients at very high relapse risk in NPM-ALK-positive anaplastic large-cell lymphoma. Blood. 2014;123(3):334-7.

9. Beldjord K, Chevret S, Asnafi V, Huguet F, Boulland ML, Leguay T, et al. Oncogenetics and minimal residual disease are independent outcome predictors in adult patients with acute lymphoblastic leukemia. Blood. 2014;123(24):3739-49.

10. Hubmann M, Kohnke T, Hoster E, Schneider S, Dufour A, Zellmeier E, et al. Molecular response assessment by quantitative real-time polymerase chain reaction after induction therapy in NPM1-mutated patients identifies those at high risk of relapse. Haematologica. 2014;99(8):1317-25.

11. Wang Y, Wu DP, Liu QF, Qin YZ, Wang JB, Xu LP, et al. In adults with t(8;21)AML, posttransplant RUNX1/RUNX1T1-based MRD monitoring, rather than c-KIT mutations, allows further risk stratification. Blood. 2014;124(12):1880-6.

12. Pulsipher MA, Langholz B, Wall DA, Schultz KR, Bunin N, Carroll WL, et al. The addition of sirolimus to tacrolimus/methotrexate GVHD prophylaxis in children with ALL: a phase 3 Children's Oncology Group/Pediatric Blood and Marrow Transplant Consortium trial. Blood. 2014;123(13):2017-25.

13. Eckert C, Hagedorn N, Sramkova L, Mann G, Panzer-Grumayer R, Peters C, et al. Monitoring minimal residual disease in children with high-risk relapses of acute lymphoblastic leukemia: prognostic relevance of early and late assessment. Leukemia. 2015;29(8):1648-55.

14. Pulsipher MA, Carlson C, Langholz B, Wall DA, Schultz KR, Bunin N, et al. IgH-V(D)J NGS-MRD measurement pre- and early post-allotransplant defines very low- and very high-risk ALL patients. Blood. 2015;125(22):3501-8.

15. Bader P, Kreyenberg H, von Stackelberg A, Eckert C, Salzmann-Manrique E, Meisel R, et al. Monitoring of minimal residual disease after allogeneic stem-cell transplantation in relapsed childhood acute lymphoblastic leukemia allows for the identification of impending relapse: results of the ALL-BFM-SCT 2003 trial. J Clin Oncol. 2015;33(11):1275-84.

16. Chen X, Xie H, Wood BL, Walter RB, Pagel JM, Becker PS, et al. Relation of clinical response and minimal residual disease and their prognostic impact on outcome in acute myeloid leukemia. J Clin Oncol. 2015;33(11):1258-64.

17. Ivey A, Hills RK, Simpson MA, Jovanovic JV, Gilkes A, Grech A, et al. Assessment of Minimal Residual Disease in Standard-Risk AML. N Engl J Med. 2016;374(5):422-33.

18. Willekens C, Blanchet O, Renneville A, Cornillet-Lefebvre P, Pautas C, Guieze R, et al. Prospective long-term minimal residual disease monitoring using RQ-PCR in RUNX1-RUNX1T1-positive acute myeloid leukemia: results of the French CBF-2006 trial. Haematologica. 2016;101(3):328-35.

19. Araki D, Wood BL, Othus M, Radich JP, Halpern AB, Zhou Y, et al. Allogeneic Hematopoietic Cell Transplantation for Acute Myeloid Leukemia: Time to Move Toward a Minimal Residual Disease-Based Definition of Complete Remission? J Clin Oncol. 2016;34(4):329-36.

20. Zeijlemaker W, Kelder A, Oussoren-Brockhoff YJ, Scholten WJ, Snel AN, Veldhuizen D, et al. Peripheral blood minimal residual disease may replace bone marrow minimal residual disease as an immunophenotypic biomarker for impending relapse in acute myeloid leukemia. Leukemia. 2016;30(3):708-15.

21. Zhou Y, Othus M, Araki D, Wood BL, Radich JP, Halpern AB, et al. Pre- and post-transplant quantification of measurable ('minimal') residual disease via multiparameter flow cytometry in adult acute myeloid leukemia. Leukemia. 2016;30(7):1456-64.

22. Oran B, Jorgensen JL, Marin D, Wang S, Ahmed S, Alousi AM, et al. Pre-transplantation minimal residual disease with cytogenetic and molecular diagnostic features improves risk stratification in acute myeloid leukemia. Haematologica. 2017;102(1):110-7.

23. Guolo F, Minetto P, Clavio M, Miglino M, Galaverna F, Raiola AM, et al. Combining flow cytometry and WT1 assessment improves the prognostic value of pre-transplant minimal residual disease in acute myeloid leukemia. Haematologica. 2017;102(9):e348-e51.

24. Balsat M, Renneville A, Thomas X, de Botton S, Caillot D, Marceau A, et al. Postinduction Minimal Residual Disease Predicts Outcome and Benefit From Allogeneic Stem Cell Transplantation in Acute Myeloid Leukemia With NPM1 Mutation: A Study by the Acute Leukemia French Association Group. J Clin Oncol. 2017;35(2):185-93.

25. Gaballa S, Saliba R, Oran B, Brammer JE, Chen J, Rondon G, et al. Relapse risk and survival in patients with FLT3 mutated acute myeloid leukemia undergoing stem cell transplantation. Am J Hematol. 2017;92(4):331-7.

26. Chang YJ, Zhao XS, Wang Y, Liu YR, Xu LP, Zhang XH, et al. Effects of pre- and post-transplantation minimal residual disease on outcomes in pediatric patients with acute myeloid leukemia receiving human leukocyte antigen-matched or mismatched related donor allografts. Am J Hematol. 2017;92(12):E659-E61.

27. Boddu P, Jorgensen J, Kantarjian H, Borthakur G, Kadia T, Daver N, et al. Achievement of a negative minimal residual disease state after hypomethylating agent therapy in older patients with AML reduces the risk of relapse. Leukemia. 2018;32(1):241-4.

28. Cazzaniga G, De Lorenzo P, Alten J, Rottgers S, Hancock J, Saha V, et al. Predictive value of minimal residual disease in Philadelphia-chromosome-positive acute lymphoblastic leukemia treated with imatinib in the European intergroup study of post-induction treatment of Philadelphia-chromosome-positive acute lymphoblastic leukemia, based on immunoglobulin/T-cell receptor and BCR/ABL1 methodologies. Haematologica. 2018;103(1):107-15.

29. Jongen-Lavrencic M, Grob T, Hanekamp D, Kavelaars FG, Al Hinai A, Zeilemaker A, et al. Molecular Minimal Residual Disease in Acute Myeloid Leukemia. N Engl J Med. 2018;378(13):1189-99.

30. Morita K, Kantarjian HM, Wang F, Yan Y, Bueso-Ramos C, Sasaki K, et al. Clearance of Somatic Mutations at Remission and the Risk of Relapse in Acute Myeloid Leukemia. J Clin Oncol. 2018;36(18):1788-97.

31. Petit A, Trinquand A, Chevret S, Ballerini P, Cayuela JM, Grardel N, et al. Oncogenetic mutations combined with MRD improve outcome prediction in pediatric T-cell acute lymphoblastic leukemia. Blood. 2018;131(3):289-300.

32. Freeman SD, Hills RK, Virgo P, Khan N, Couzens S, Dillon R, et al. Measurable Residual Disease at Induction Redefines Partial Response in Acute Myeloid Leukemia and Stratifies Outcomes in Patients at Standard Risk Without NPM1 Mutations. J Clin Oncol. 2018;36(15):1486-97.

33. Thol F, Gabdoulline R, Liebich A, Klement P, Schiller J, Kandziora C, et al. Measurable residual disease monitoring by NGS before allogeneic hematopoietic cell transplantation in AML. Blood. 2018;132(16):1703-13.

34. Zhao XS, Liu YR, Xu LP, Wang Y, Zhang XH, Chen H, et al. Minimal residual disease status determined by multiparametric flow cytometry pretransplantation predicts the outcome of patients with ALL receiving unmanipulated haploidentical allografts. Am J Hematol. 2019;94(5):512-21.

35. Zeijlemaker W, Grob T, Meijer R, Hanekamp D, Kelder A, Carbaat-Ham JC, et al. CD34(+)CD38(-) leukemic stem cell frequency to predict outcome in acute myeloid leukemia. Leukemia. 2019;33(5):1102-12.

36. Rucker FG, Agrawal M, Corbacioglu A, Weber D, Kapp-Schwoerer S, Gaidzik VI, et al. Measurable residual disease monitoring in acute myeloid leukemia with t(8;21)(q22;q22.1): results from the AML Study Group. Blood. 2019;134(19):1608-18.

37. Modvig S, Madsen HO, Siitonen SM, Rosthoj S, Tierens A, Juvonen V, et al. Minimal residual disease quantification by flow cytometry provides reliable risk stratification in T-cell acute lymphoblastic leukemia. Leukemia. 2019;33(6):1324-36.

38. Morsink LM, Othus M, Bezerra ED, Wood BL, Fang M, Sandmaier BM, et al. Impact of pretransplant measurable residual disease on the outcome of allogeneic hematopoietic cell transplantation in adult monosomal karyotype AML. Leukemia. 2020;34(6):1577-87.

39. Baron F, Labopin M, Ruggeri A, Sierra J, Robinson S, Labussiere-Wallet H, et al. Impact of detectable measurable residual disease on umbilical cord blood transplantation. Am J Hematol. 2020;95(9):1057-65.

40. Paiva B, Puig N, Cedena MT, Rosinol L, Cordon L, Vidriales MB, et al. Measurable Residual Disease by Next-Generation Flow Cytometry in Multiple Myeloma. J Clin Oncol. 2020;38(8):784-92.

41. Perez-Martinez A, Ferreras C, Pascual A, Gonzalez-Vicent M, Alonso L, Badell I, et al. Haploidentical transplantation in high-risk pediatric leukemia: A retrospective comparative analysis on behalf of the Spanish working Group for bone marrow transplantation in children (GETMON) and the Spanish Grupo for hematopoietic transplantation (GETH). Am J Hematol. 2020;95(1):28-37.

42. Zhou B, Chu X, Tian H, Liu T, Liu H, Gao W, et al. The clinical outcomes and genomic landscapes of acute lymphoblastic leukemia patients with E2A-PBX1: A 10-year retrospective study. Am J Hematol. 2021;96(11):1461-71.

43. Paiva B, Vidriales MB, Sempere A, Tarin F, Colado E, Benavente C, et al. Impact of measurable residual disease by decentralized flow cytometry: a PETHEMA real-world study in 1076 patients with acute myeloid leukemia. Leukemia. 2021;35(8):2358-70.

44. Cho BS, Min GJ, Park S, Park SS, Shin SH, Yahng SA, et al. Haploidentical vs matched unrelated donor transplantation for acute myeloid leukemia in remission: A prospective comparative study. Am J Hematol. 2021;96(1):98-109.

45. Sidhom I, Shaaban K, Youssef SH, Ali N, Gohar S, Rashed WM, et al. Reduced-intensity therapy for pediatric lymphoblastic leukemia: impact of residual disease early in remission induction. Blood. 2021;137(1):20-8.

46. Esteve J, Giebel S, Labopin M, Czerw T, Wu D, Volin L, et al. Allogeneic hematopoietic stem cell transplantation for adult patients with t(4;11)(q21;q23) KMT2A/AFF1 B-cell precursor acute lymphoblastic leukemia in first complete remission: impact of pretransplant measurable residual disease (MRD) status. An analysis from the Acute Leukemia Working Party of the EBMT. Leukemia. 2021;35(8):2232-42.

47. Jentzsch M, Grimm J, Bill M, Brauer D, Backhaus D, Pointner R, et al. Clinical value of the measurable residual disease status within the ELN2017 risk groups in AML patients undergoing allogeneic stem cell transplantation. Am J Hematol. 2021;96(7):E237-E9.

48. den Boer ML, Cario G, Moorman AV, Boer JM, de Groot-Kruseman HA, Fiocco M, et al. Outcomes of paediatric patients with B-cell acute lymphocytic leukaemia with ABL-class fusion in the pre-tyrosine-kinase inhibitor era: a multicentre, retrospective, cohort study. Lancet Haematol. 2021;8(1):e55-e66.

49. Patkar N, Kakirde C, Shaikh AF, Salve R, Bhanshe P, Chatterjee G, et al. Clinical impact of panel-based error-corrected next generation sequencing versus flow cytometry to detect measurable residual disease (MRD) in acute myeloid leukemia (AML). Leukemia. 2021;35(5):1392-404.

50. Puckrin R, Atenafu EG, Claudio JO, Chan S, Gupta V, Maze D, et al. Measurable residual disease monitoring provides insufficient lead-time to prevent morphologic relapse in the majority of patients with core-binding factor acute myeloid leukemia. Haematologica. 2021;106(1):56-63.

51. Modvig S, Hallbook H, Madsen HO, Siitonen S, Rosthoj S, Tierens A, et al. Value of flow cytometry for MRD-based relapse prediction in B-cell precursor ALL in a multicenter setting. Leukemia. 2021;35(7):1894-906.

52. Popov A, Henze G, Roumiantseva J, Budanov O, Belevtsev M, Verzhbitskaya T, et al. A simple algorithm with one flow cytometric MRD measurement identifies more than 40% of children with ALL who can be cured with low-intensity therapy. The ALL-MB 2008 trial results. Leukemia. 2022;36(5):1382-5.

53. Marks DI, Clifton-Hadley L, Copland M, Hussain J, Menne TF, McMillan A, et al. In-vivo T-cell depleted reduced-intensity conditioned allogeneic haematopoietic stem-cell transplantation for patients with acute lymphoblastic leukaemia in first remission: results from the prospective, single-arm evaluation of the UKALL14 trial. Lancet Haematol. 2022;9(4):e276-e88.

54. Escherich G, Zur Stadt U, Borkhardt A, Dilloo D, Faber J, Feuchtinger T, et al. Clofarabine increases the eradication of minimal residual disease of primary B-precursor acute lymphoblastic leukemia compared to high-dose cytarabine without improvement of outcome. Results from the randomized clinical trial 08-09 of the Cooperative Acute Lymphoblastic Leukemia Study Group. Haematologica. 2022;107(5):1026-33.

55. Paras G, Morsink LM, Othus M, Milano F, Sandmaier BM, Zarling LC, et al. Conditioning intensity and peritransplant flow cytometric MRD dynamics in adult AML. Blood. 2022;139(11):1694-706.

56. Li SQ, Xu LP, Wang Y, Zhang XH, Chen H, Chen YH, et al. An LSC-based MRD assay to complement the traditional MFC method for prediction of AML relapse: a prospective study. Blood. 2022;140(5):516-20.

57. Attarbaschi A, Moricke A, Harrison CJ, Mann G, Baruchel A, De Moerloose B, et al. Outcomes of Childhood Noninfant Acute Lymphoblastic Leukemia With 11q23/KMT2A Rearrangements in a Modern Therapy Era: A Retrospective International Study. J Clin Oncol. 2023;41(7):1404-22.

58. Wood B, Devidas M, Summers RJ, Chen Z, Asselin BL, Rabin KR, et al. Prognostic Significance of ETP Phenotype and Minimal Residual Disease in T-ALL: A Children's Oncology Group Study. Blood. 2023;142(24):2069-78.

59. Orvain C, Wilson JA, Fang M, Sandmaier BM, Rodriguez-Arboli E, Wood BL, et al. Relative impact of residual cytogenetic abnormalities and flow cytometric measurable residual disease on outcome after allogeneic hematopoietic cell transplantation in adult acute myeloid leukemia. Haematologica. 2023;108(2):420-32.

60. Dillon LW, Gui G, Page KM, Ravindra N, Wong ZC, Andrew G, et al. DNA Sequencing to Detect Residual Disease in Adults With Acute Myeloid Leukemia Prior to Hematopoietic Cell Transplant. JAMA. 2023;329(9):745-55.

61. Othman J, Tiong IS, O'Nions J, Dennis M, Mokretar K, Ivey A, et al. Molecular MRD is strongly prognostic in patients with NPM1-mutated AML receiving venetoclax-based non-intensive therapy. Blood. 2023;143(4):336-41.

62. Dillon LW, Higgins J, Nasif H, Othus M, Beppu L, Smith TH, et al. Quantification of measurable residual disease using duplex sequencing in adults with acute myeloid leukemia. Haematologica. 2024;109(2):401-10.

63. Pasvolsky O, Saliba RM, Ledesma C, Popat UR, Alousi A, Olson A, et al. Prognostic significance of measurable residual disease in patients with acute lymphoblastic leukemia undergoing allogeneic hematopoietic stem cell transplantation in second or later complete remission. Am J Hematol. 2023;98(2):E35-E7.

64. Kim R, Bergugnat H, Pastoret C, Pasquier F, Raffoux E, Larcher L, et al. Genetic alterations and MRD refine risk assessment for KMT2A-rearranged B-cell precursor ALL in adults: a GRAALL study. Blood. 2023;142(21):1806-17.

65. van Weelderen RE, Klein K, Harrison CJ, Jiang Y, Abrahamsson J, Arad-Cohen N, et al. Measurable Residual Disease and Fusion Partner Independently Predict Survival and Relapse Risk in Childhood KMT2A-Rearranged Acute Myeloid Leukemia: A Study by the International Berlin-Frankfurt-Munster Study Group. J Clin Oncol. 2023;41(16):2963-74.

66. Grob T, Sanders MA, Vonk CM, Kavelaars FG, Rijken M, Hanekamp DW, et al. Prognostic Value of FLT3-Internal Tandem Duplication Residual Disease in Acute Myeloid Leukemia. J Clin Oncol. 2023;41(4):756-65.

67. Garcia-Murillas I, Chopra N, Comino-Mendez I, Beaney M, Tovey H, Cutts RJ, et al. Assessment of Molecular Relapse Detection in Early-Stage Breast Cancer. JAMA Oncol. 2019;5(10):1473-8.

68. Christensen E, Birkenkamp-Demtroder K, Sethi H, Shchegrova S, Salari R, Nordentoft I, et al. Early Detection of Metastatic Relapse and Monitoring of Therapeutic Efficacy by Ultra-Deep Sequencing of Plasma Cell-Free DNA in Patients With Urothelial Bladder Carcinoma. J Clin Oncol. 2019;37(18):1547-57.

69. Wang Y, Li L, Cohen JD, Kinde I, Ptak J, Popoli M, et al. Prognostic Potential of Circulating Tumor DNA Measurement in Postoperative Surveillance of Nonmetastatic Colorectal Cancer. JAMA Oncol. 2019;5(8):1118-23.

70. Tan L, Sandhu S, Lee RJ, Li J, Callahan J, Ftouni S, et al. Prediction and monitoring of relapse in stage III melanoma using circulating tumor DNA. Ann Oncol. 2019;30(5):804-14.

71. Reinert T, Henriksen TV, Christensen E, Sharma S, Salari R, Sethi H, et al. Analysis of Plasma Cell-Free DNA by Ultradeep Sequencing in Patients With Stages I to III Colorectal Cancer. JAMA Oncol. 2019;5(8):1124-31.

72. Tie J, Cohen JD, Wang Y, Christie M, Simons K, Lee M, et al. Circulating Tumor DNA Analyses as Markers of Recurrence Risk and Benefit of Adjuvant Therapy for Stage III Colon Cancer. JAMA Oncol. 2019;5(12):1710-7.

73. Radovich M, Jiang G, Hancock BA, Chitambar C, Nanda R, Falkson C, et al. Association of Circulating Tumor DNA and Circulating Tumor Cells After Neoadjuvant Chemotherapy With Disease Recurrence in Patients With Triple-Negative Breast Cancer: Preplanned Secondary Analysis of the BRE12-158 Randomized Clinical Trial. JAMA Oncol. 2020;6(9):1410-5.

74. Magbanua MJM, Swigart LB, Wu HT, Hirst GL, Yau C, Wolf DM, et al. Circulating tumor DNA in neoadjuvant-treated breast cancer reflects response and survival. Ann Oncol. 2021;32(2):229-39.

75. Ococks E, Frankell AM, Masque Soler N, Grehan N, Northrop A, Coles H, et al. Longitudinal tracking of 97 esophageal adenocarcinomas using liquid biopsy sampling. Ann Oncol. 2021;32(4):522-32.

76. Lipsyc-Sharf M, de Bruin EC, Santos K, McEwen R, Stetson D, Patel A, et al. Circulating Tumor DNA and Late Recurrence in High-Risk Hormone Receptor-Positive, Human Epidermal Growth Factor Receptor 2-Negative Breast Cancer. J Clin Oncol. 2022;40(22):2408-19.

77. Gale D, Heider K, Ruiz-Valdepenas A, Hackinger S, Perry M, Marsico G, et al. Residual ctDNA after treatment predicts early relapse in patients with early-stage non-small cell lung cancer. Ann Oncol. 2022;33(5):500-10.

78. Kotani D, Oki E, Nakamura Y, Yukami H, Mishima S, Bando H, et al. Molecular residual disease and efficacy of adjuvant chemotherapy in patients with colorectal cancer. Nat Med. 2023;29(1):127-34.

79. Mo S, Ye L, Wang D, Han L, Zhou S, Wang H, et al. Early Detection of Molecular Residual Disease and Risk Stratification for Stage I to III Colorectal Cancer via Circulating Tumor DNA Methylation. JAMA Oncol. 2023;9(6):770-8.

80. Galbraith RF. A note on graphical presentation of estimated odds ratios from several clinical trials. Stat Med. 1988;7(8):889-94.

81. Egger M, Davey Smith G, Schneider M, Minder C. Bias in meta-analysis detected by a simple, graphical test. BMJ. 1997;315(7109):629-34.

82. Prentice RL. Surrogate endpoints in clinical trials: definition and operational criteria. Stat Med. 1989;8(4):431-40.
